# Screening for mutations in human cardiomyopathy- is *RBM24* a new but rare disease gene?

**DOI:** 10.1007/s13238-018-0590-z

**Published:** 2018-11-12

**Authors:** Anna Gaertner, Andreas Brodehl, Hendrik Milting

**Affiliations:** Erich and Hanna Klessmann Institute, Heart and Diabetes Center NRW, University Hospital of the Ruhr-University Bochum, Georgstrasse 11, 32545 Bad Oeynhausen, Germany

With great interest we have read the study of Liu et al. (2018) revealing the role of RNA binding protein 24 (RBM24) on global alternative splicing and dilated cardiomyopathy (DCM) in mice. As suggested previously, deficiency of *Rbm24* causes embryonic lethality limiting the functional analyses (Yang et al., 2014). To circumvent this limitation the authors generated cardiac specific *Rbm24* deficient mice and showed that homozygous deletion of *Rbm24* at postnatal stage leads to rapidly progressive DCM and heart failure (Liu et al., 2018).

DCM is considered as the most common type of cardiomyopathy (Fu and Eisen, 2018). It is estimated that about 60% of DCM cases have a genetic etiology (Klauke et al., 2017). However, the associated genotypes are highly diverse and mutations in at least 50 genes have been described with DCM (Stenson et al., 2003). These genes are encoding proteins of the sarcomere, the cytoskeleton, the nuclear envelope, components of the Ca^2+^ transient, ion channels and transcription (co)-factors. In addition, several regulators of alternative splicing like muscleblind like splicing regulator 1 (MBNL1) (LeMasters et al., 2012), RNA binding fox-1 homolog 1 (RBFOX1) (Gao et al., 2016), RNA binding fox-1 homolog 2 (RBFOX2) (Wei et al., 2015), RBM24 (Liu et al., 2018) might have impact on cardiac development and/or the cardiac function in animal models. However, the role of these proteins in human cardiomyopathy is still unknown. At present, only mutations in the gene *RBM20* encoding a cardiac splice factor have been shown to be associated with cardiomyopathy (Brauch et al., 2009; Wells et al., 2013; Beqqali et al., 2016).

Conditional deletion of *Rbm24* is associated with 590 altered splicing events in mice. The affected proteins are involved in cytoskeleton organization, striated muscle cell differentiation, heart contraction and cardiovascular development (Liu et al., 2018). Interestingly, the study of Liu et al. demonstrated convincingly that one of the direct splicing targets of murine Rbm24 is *Ttn,* coding for the giant sarcomeric protein titin. RNA binding protein 20 (RBM20) was the only known splicing factor of *TTN* (Guo et al., 2012). Remarkably, mutations in *RBM20* are not so rare among DCM-patients and cause frequently a severe clinical phenotype (Brauch et al., 2009; Ma et al., 2016).

We think that the study of Liu et al. might has also relevance for human cardiovascular genetics because it can be suggested that *RBM24* mutations might be involved in human cardiomyopathies, especially because no disease associated *RBM24*-mutations have been described until now.

Even if familial cardiomyopathy is suspected, the identification of the causative pathogenic gene mutation is not always possible. In consequence, the identification of novel cardiomyopathy associated genes remains a major issue of human cardiovascular genetics having clinical impact for genetic counselling of affected families.

As RBM24 was already established as a major regulator of muscle-specific alternative splicing (Yang et al., 2014) we analyzed the coding sequence of three *RBM24-*splice variants (NM_001143942, NM_153020, NM_001143941) in 190 cardiomyopathy index patients by Sanger sequencing. To our surprise, we did not find any pathogenic *RBM24* mutation. Although we cannot exclude that in further specific cases pathogenic *RBM24* mutations might be found, our data suggest that *RBM24* mutations are rare in human cardiomyopathy patients. Thus, the data of our cohort suggests an allele frequency in non-ischemic cardiomyopathy patients below <0.5%. These findings are supported by the fact that only the homozygous *Rbm24* deficient mice developed DCM in the study by Liu and colleagues (2018).

In summary, it remains an open question if *RBM24* can be established as a rare cardiomyopathy associated gene. However, with relevance for clinical cardiovascular genetics this gene deserves further attention in genetic screenings.

## References

[CR1] Beqqali A, Bollen IA, Rasmussen TB, van den Hoogenhof MM, van Deutekom HW, Schafer S, Haas J, Meder B, Sørensen KE, van Oort RJ (2016). A mutation in the glutamate-rich region of RNA-binding motif protein 20 causes dilated cardiomyopathy through missplicing of titin and impaired Frank-Starling mechanism. Cardiovasc Res.

[CR2] Brauch KM, Karst ML, Herron KJ, de Andrade M, Pellikka PA, Rodeheffer RJ, Michels VV, Olson TM (2009). Mutations in ribonucleic acid binding protein gene cause familial dilated cardiomyopathy. J Am Coll Cardiol.

[CR3] Fu Y, Eisen HJ (2018). Genetics of Dilated Cardiomyopathy. Curr Cardiol Rep.

[CR4] Gao C, Ren S, Lee JH, Qiu J, Chapski DJ, Rau CD, Zhou Y, Abdellatif M, Nakano A, Vondriska TM (2016). RBFox1-mediated RNA splicing regulates cardiac hypertrophy and heart failure. J Clin Invest.

[CR5] Guo W, Schafer S, Greaser ML, Radke MH, Liss M, Govindarajan T, Maatz H, Schulz H, Li S, Parrish AM (2012). RBM20, a gene for hereditary cardiomyopathy, regulates titin splicing. Nat Med.

[CR6] Klauke B, Gaertner-Rommel A, Schulz U, zu Kassner A, Knyphausen E, Laser T, Kececioglu D, Paluszkiewicz L, Blanz U, Sandica E (2017). High proportion of genetic cases in patients with advanced cardiomyopathy including a novel homozygous Plakophilin 2-gene mutation. PLoS ONE.

[CR7] LeMasters KE, Blech-Hermoni Y, Stillwagon SJ, Vajda NA, Ladd AN (2012). Loss of muscleblind-like 1 promotes invasive mesenchyme formation in endocardial cushions by stimulating autocrine TGFbeta3. BMC Dev Biol.

[CR8] Liu J, Kong X, Zhang M, Yang X, Xu X (2018). RNA binding protein 24 deletion disrupts global alternative splicing and causes dilated cardiomyopathy. Protein Cell.

[CR9] Ma J, Lu L, Guo W, Ren J, Yang J (2016). Emerging role for RBM20 and its splicing substrates in cardiac function and heart failure. Curr Pharm Des.

[CR10] Stenson PD, Ball EV, Mort M, Phillips AD, Shiel JA, Thomas NS, Abeysinghe S, Krawczak M, Cooper DN (2003). Human Gene Mutation Database (HGMD): 2003 update. Hum Mutat.

[CR11] Wei C, Qiu J, Zhou Y, Xue Y, Hu J, Ouyang K, Banerjee I, Zhang C, Chen B, Li H (2015). Repression of the central splicing regulator RBFox2 is functionally linked to pressure overload-induced heart failure. Cell Rep.

[CR12] Wells QS, Becker JR, Su YR, Mosley JD, Weeke P, Daoust L, Ausborn NL, Ramirez AH, Pfotenhauer JP, Naftilan AJ (2013). Whole exome sequencing identifies a causal RBM20 mutation in a large pedigree with familial dilated cardiomyopathy. Circ Cardiovasc Genet.

[CR13] Yang J, Hung LH, Licht T, Kostin S, Looso M, Khrameeva E, Bindereif A, Schneider A, Braun T (2014). RBM24 is a major regulator of muscle-specific alternative splicing. Dev Cell.

